# Centhaquine Increases Stroke Volume and Cardiac Output in Patients with Hypovolemic Shock

**DOI:** 10.3390/jcm13133765

**Published:** 2024-06-27

**Authors:** Aman Khanna, Krish Vaidya, Dharmesh Shah, Amaresh K. Ranjan, Anil Gulati

**Affiliations:** 1 Aman Hospital and Research Centre Organization, Vadodara 390021, GJ, India; amankhanna1974@gmail.com; 2 I Cure Heart Care, Vadodara 390007, GJ, India; vaidya.krish87@gmail.com; 3 Pharmazz India Private Limited, Greater Noida 201307, UP, India; dharmesh.shah@pharmazz.com; 4 Pharmazz Inc., Research and Development, Willowbrook, IL 60527, USA; 5 Department of Bioengineering, The University of Illinois at Chicago, Chicago, IL 60607, USA; 6 College of Pharmacy Downers Grove, Midwestern University, Downers Grove, IL 60515, USA

**Keywords:** centhaquine, cardiac output, venous return, blood pressure, shock, hypovolemia, resuscitation, critical care

## Abstract

**Introduction:** Centhaquine is a resuscitative agent that acts on α2B adrenergic receptors. Its effect on cardiac output in hypovolemic shock patients has not been reported. **Methods:** This pilot study was conducted in 12 hypovolemic shock patients treated with centhaquine who participated in an open-label phase IV study (NCT05956418). Echocardiography was utilized to measure stroke volume (SV), cardiac output (CO), left ventricular outflow tract velocity time integral (LVOT-VTI) and diameter (LVOTd), heart rate (HR), left ventricular ejection fraction (LVEF) and fractional shortening (LVFS), and inferior vena cava (IVC) diameter before (0 min) and 60, 120, and 300 min after centhaquine (0.01 mg/kg) iv infusion for 60 min. **Results**: SV was significantly increased after 60, 120, and 300 min. CO increased significantly after 120 and 300 min despite a decrease in HR. IVC diameter and LVOT-VTI at these time points significantly increased, indicating the increased venous return. LVEF and LVFS did not change, while the mean arterial pressure (MAP, mmHg) increased after 120 and 300 min. Positive correlations between IVC diameter and SV (R^2^ = 0.9556) and between IVC diameter and MAP (R^2^ = 0.8928) were observed, which indicated the effects of an increase in venous return on SV, CO, and MAP. **Conclusions**: Centhaquine-mediated increase in venous return is critical in enhancing SV, CO, and MAP in patients with hypovolemic shock; these changes could be pivotal for reducing shock-mediated circulatory failure, promoting tissue perfusion, and improving patient outcomes. **Trial Registration**: CTRI/2021/01/030263 and NCT05956418.

## 1. Introduction

Hypovolemic shock decreases circulating blood volume and reduces stroke volume (SV), cardiac output (CO) [1], and tissue blood perfusion, leading to the possibility of organ failure and death. It is vital to initiate treatment to restore the affected hemodynamics and to prevent the advancement from reversible organ dysfunction to irreversible multiorgan failure following shock. The current management strategy broadly includes fluid therapy to expand intravascular space, blood products, ventilation for oxygen support, and vasopressors to counter hypotension and increase tissue perfusion [1,2]. Adequate fluid resuscitation is essential since errors in volume administration can result in either a lack of essential treatment or unnecessary fluid administration (fluid overload), and both scenarios are associated with poor patient outcomes [3]. Nonetheless, achieving an appropriate volume level is challenging and requires an understanding of the underlying pathophysiology, volume and tissue perfusion statuses, and fluid volume responsiveness [4,5,6]. Thus, a frequent assessment of fluid volume responsiveness as well as volume status in patients with shock is important for improving tissue perfusion and avoiding volume overload [4,5,6]. According to Refs. [1,2], although the commonly used fluids (crystalloids or colloids) help compensate for the volume loss, patients often require vasopressors to achieve perfusion endpoints (e.g., CO and MAP) [7]. Vasopressors induce constriction of the blood vessels and aid the sympathetic system to increase blood pressure and CO in an attempt to increase tissue perfusion [8]. However, the use of vasopressors (e.g., epinephrine, norepinephrine, dopamine, vasopressin, and angiotensin) remains debatable due to associated risks, including cardiac arrhythmias, decreased tissue perfusion, fluid extravasation, and organ failure [9].

In the normal physiological state, a significant amount of blood is pooled on the venous side (having high vascular capacitance) of the circulation; however, in hypovolemic shock, blood accumulation in the veins is further increased, and a significant amount of blood does not participate in tissue perfusion [10]. It is of interest to divert the pooled venous blood toward the arterial side so that the circulating blood volume can be increased, which will help increase stroke volume (SV), cardiac output (CO), and mean arterial pressure (MAP), as well as increase tissue perfusion.

Centhaquine (2-[2-[4-(3-Methylphenyl)-1-piperazinyl]ethyl] quinoline citrate) is being developed to treat hypovolemic shock. It showed good tolerability and safety in healthy subjects with minimal adverse effects observed at almost ten times the therapeutic dose (NCT02408731). Clinical studies in hypovolemic shock patients (NCT04056065 and NCT04045327) found that centhaquine significantly increased MAP, reduced lactate levels, and increased survival [11,12]. The proposed mechanism of action of centhaquine is by acting on venous α2B adrenergic receptors; it augments the blood return to the heart and increases SV [13]. In swine and rat models of hypovolemic shock, centhaquine significantly increased SV, leading to increased CO and MAP [14] and improved survival. The current study investigates centhaquine’s role in increasing cardiovascular variables (SV, CO, and MAP) by enhancing venous blood return in human patients with hypovolemic shock.

## 2. Materials and Methods

### 2.1. Study Design

This pilot study was conducted on 12 patients participating in an open-label phase IV clinical study (NCT05956418) of centhaquine in patients with hypovolemic shock receiving the best SOC. The study was conducted at Aman Hospital and Research Centre, 15 Shashwat, Opp, E.S.I Hospital Sarabhai, Gotri Road, Vadodara, Gujarat-390021, India. At the baseline, demographic data, chest X-ray, electrocardiogram (ECG), and vital signs were recorded along with blood counts and chemistry. Patients received 0.01 mg/kg centhaquine through intravenous infusion along with SOC (e.g., airway maintenance, fluid resuscitation with crystalloids/colloids) for hypovolemic shock. Centhaquine (0.01 mg/kg body wt) was infused over 1 h in 100 mL of normal saline. Systolic and diastolic blood pressure (SBP and DBP) were recorded using a sphygmomanometer at baseline, hourly for the initial 48 h, and in between if needed. The MAP [DBP + 1/3 (SBP − DBP)] and pulse pressure (SBP-DBP) were calculated. Trans-thoracic echocardiography was utilized to assess SV, CO, HR, left ventricular ejection fraction (LVEF), and left ventricular fractional shortening (LVFS) before (0 min) and after centhaquine treatment (60 min, 120 min, and 300 min). 

All patients in this study received centhaquine along with standard of care for hypovolemic shock. The study duration for an individual patient was seven days or less (patient discharge). Data from the baseline value in this study were compared. Historical data on fluid resuscitation, as well as data from a previously conducted phase II trial (NCT04056065) of centhaquine in patients with hypovolemic shock, were used to indicate the role of centhaquine in cardiovascular improvement following shock.

#### 2.1.1. Echocardiographic Measurements

Transthoracic echocardiography was utilized to assess SV, CO, HR, LVEF, and LVFS before (0 min) and after centhaquine treatment (60 min, 120 min, and 300 min). An expert technician conducted the echocardiography, and the detailed methodology is as follows.

##### LVOT Diameter

A parasternal long-axis view was obtained to visualize the left ventricular outflow tract (LVOT) and the aortic valve. A view of the aortic valve opening and closing was ensured. The best view of the aortic valve at mid-systole (when the valves are wide open) was ensured, images were captured, and the distance near the aortic annulus at the base of the leaflets was measured using the tool. This measured distance was the diameter of the LVOT.

##### LVOT VTI (LVOT Velocity Time Integral)

The pulse wave Doppler gate was aligned parallel to the LVOT in the Apical 5 Chamber view to obtain the best VTI tracing. Once the Doppler gate was positioned well, the pulse wave Doppler was activated. The automated LVOT VTI was calculated after tracing the outline of one of the systolic waveforms. The values for the LVOT VTI are denoted as the distance in centimeters (cm) and represent the distance that blood travels in one heartbeat.

##### Heart Rate (HR)

The specific points on the screen corresponding to individual heartbeats were marked, and the heart rate was automatically calculated by the echocardiography machine based on this input.

##### SV and CO

SV was calculated as the product of the LVOT diameter (LVOTd) and the LVOT VTI (SV = LVOTd × LVOT VTI). CO was calculated as the product of SV and HR (CO = SV × HR).

##### LVEF and LVFS

LVEF was calculated via visual estimation by reviewing different echocardiography windows, as is done in an emergency setting. LVFS was calculated using the formula %LVFS = [(LVDD − LVDS)/LVDD] × 100, where LVDD is the left ventricle diameter at diastole, and LVDS is the left ventricle diameter at systole, measured through echocardiography.

##### Total Peripheral Resistance (TPR)

TPR was calculated using the following formula: TPR = (79.9 × MAP)/CO.

### 2.2. Patient Population, Consent, and Regulatory Oversight

Patients were screened for phase IV study (NCT05956418) eligibility according to the following inclusion and exclusion criteria. Inclusion criteria—adult hypovolemic shock patients aged ≥ 18 years with an SBP ≤ 90 mmHg upon presentation to the emergency room or ICU, receiving standard shock treatment, and having a blood lactate level > 2.0 mmol/L. Exclusion criteria—patients who developed any other terminal illness not associated with hypovolemic shock during the study duration; had altered consciousness but not due to hypovolemic shock; had a known pregnancy; had undergone cardiopulmonary resuscitation (CPR) before enrollment; had not been resuscitated; participated in another interventional study; and had systemic diseases that were already present before having trauma, such as sepsis, cancer, chronic renal failure, liver failure, decompensated heart failure, or AIDS.

The patients included in this study were in a state of life-threatening shock; therefore, for patients who were not fit to give consent themselves at the time of treatment initiation, informed consent was obtained from their legally authorized representative (LAR). The investigator verbally informed the patient or the LAR as well as in writing about the details of this study relevant to a decision about whether to participate in this study.

The present study adhered to the Harmonization of Technical Requirements for Registration of Pharmaceuticals for Human Use Guideline for Good Clinical Practice (ICH-GCP), the Helsinki Declaration, and local regulatory requirements. 

This study was approved (AHRC/IEC/14/2022, approval date 30 November 2022) by the Institutional Ethics Committee Aman Hospital and Research Centre, 15 Shashwat, Opp, E.S.I Hospital Sarabhai, Gotri Road, Vadodara, Gujarat-390021, India. 

### 2.3. Safety Evaluation

The safety analysis was carried out by the study investigator for all patients included in this study. Safety was evaluated based on adverse events (AEs), physical examination results, vital signs (including HR, SBP, DBP, body temperature, and respiratory rate), ECG, and clinical variables. Any AEs that occurred or worsened during or after centhaquine treatment were systematically recorded and coded by system organ class and preferred term using the latest version of the International Conference on Harmonization Medical Dictionary for Regulatory Activities. 

### 2.4. Multivariate Imputation by Chained Equations (MICE)

Data not available (6.4%) were assessed as “missing values”, and they were imputed using “MICE”, which is a package that implements a method to address missing data by creating multiple imputations (replacement values) for multivariate missing data [15]. The MICE package contains functions for inspecting missing data patterns and imputing missing data m times, resulting in m completed data sets, diagnosing the quality of the imputed values, analyzing each completed data set, pooling the results of the repeated analyses, storing and exporting the imputed data in various formats, generating simulated incomplete data, and incorporating custom imputation methods. “MICE” generates multiple imputations for incomplete multivariate data through Gibbs sampling. The algorithm imputes an incomplete column (the target column) by generating ‘plausible’ synthetic values given other columns in the data. Each incomplete column must act as a target column and have its own specific set of predictors. The default set of predictors for a given target consists of all other columns in the data. For predictors that are incomplete themselves, the most recently generated imputations are used to complete the predictors prior to the imputation of the target column. In this study, five patients had multiple imputations for missing echocardiography data. A scalar of 20, given the number of iterations and predictive mean matching “pmm”, was used.

### 2.5. Statistical Analysis

The results are presented as the mean ± standard error of the mean (SEM). Statistical analysis was performed using GraphPad Prism 10.1.2 (GraphPad, San Diego, CA, USA). Parametric analysis was carried out using a one-way analysis of variance without assuming equal variances with a normal probability distribution. The post-hoc Tukey’s multiple comparisons test was performed to estimate the significance of differences. *p* values < 0.05 were considered to indicate statistical significance at the 95% confidence level.

## 3. Results

### 3.1. Patient Demographics, Baseline Characteristics, and the Volume of Fluid Administration during the First 5 h of Resuscitation

The demographics and baseline characteristics are shown in Table 1.

### 3.2. Centhaquine Increases Stroke Volume, Cardiac Output, and Mean Arterial Pressure

**Figure 1:**
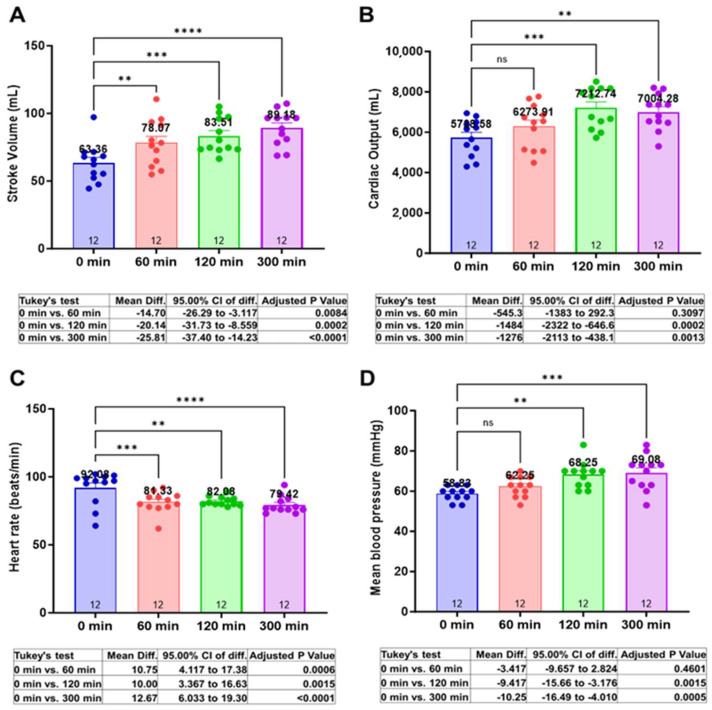
Effects of centhaquine on cardiovascular variables (SV, CO, HR, and MAP) (**A**–**D**). ** *p* < 0.01, *** *p* < 0.001, and **** *p* < 0.0001, ns—not significant compared to 0 min. *n* = 12.

At baseline (0 min), the mean SV (mL) was 63.36 ± 4.06. At 60 min, 120 min, and 300 min, the mean SV (mL) was 78.07 ± 4.98 (Δ23.2%, *p* = 0.0084), 83.51 ± 3.78 (Δ31.8%, *p* = 0.0002), and 89.18 ± 3.71 (Δ40.74%, *p* < 0.0001), respectively (Figure 1A). 

The mean CO (mL/min) at baseline was 5728.58 ± 263.4. At 60 min, 120 min, and 300 min, the mean SV (mL) was 6273.91 ± 318.33 (Δ9.52%, *p* = 0.3097), 7212.74 ± 291.2 (Δ25.9%, *p* = 0.0002), and 7004.28 ± 255.36 (Δ22.23%, *p* = 0.0013), respectively (Figure 1B).

At baseline, the mean HR (bpm) was 92.08 ± 3.55. At 60 min, 120 min, and 300 min, the mean HR (bpm) was 81.33 ± 2.23 (Δ11.69%, *p* = 0.0006), 82.1 ± 0.96 (Δ10.97%, *p* = 0.0015), and 79.42 ± 1.80 (Δ13.41%, *p* < 0.0001), respectively (Figure 1C).

MAP (mmHg) at baseline or 0 min was 58.89 ± 1.03. At 60 min, 120 min, and 300 min, the MAP values were 62.22 ±1.44 (Δ5.66%, *p* = 0.4601), 68.33 ± 1.86 (Δ16.04%, *p* = 0.0015), and 69.27 ± 2.4 (Δ17.64%, *p* = 0.0005), respectively (Figure 1D). 

### 3.3. Centhaquine Increases the Venous Return (Increase in IVC Diameter)

**Figure 2:**
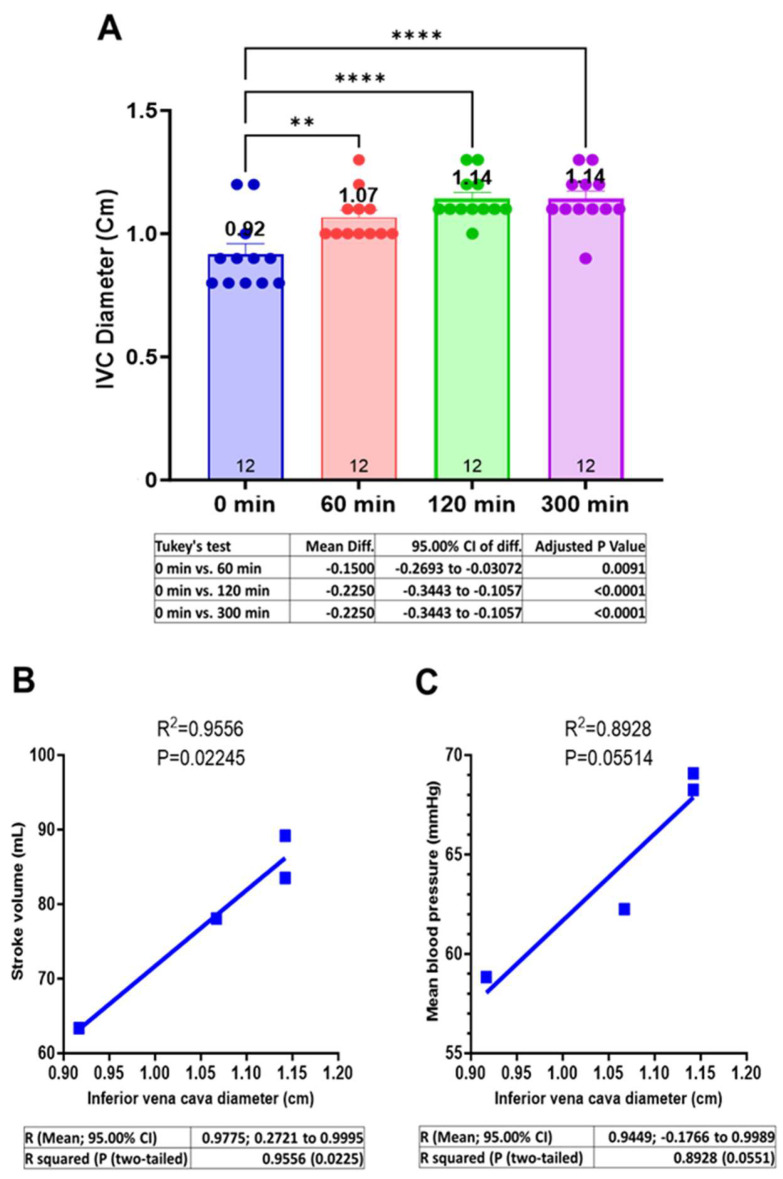
Effect of centhaquine on venous return (IVC diameter) (**A**) and its correlation with SV (**B**) and MAP (**C**), ** *p* < 0.01, and **** *p* < 0.0001 compared to 0 min. *n* = 12.

The mean IVC diameter (cm) at baseline was 0.92 ± 0.04. A change in IVC diameter was observed after centhaquine treatment at 60, 120, and 300 min; the mean IVC diameter (cm) was 1.07 ± 0.03 (Δ15.94%, *p* = 0.0091), 1.14 ± 0.02 (Δ25.00%, *p* < 0.0001), and 1.14 ± 0.03 (Δ23.19%, *p* < 0.0001), respectively (Figure 2A). 

The relationships between “IVC diameter and SV” and “IVC diameter and MAP” were evaluated. A direct correlation (R^2^ = 0.9556; *p* = 0.02245) between IVC diameter and SV (Figure 2B) and between IVC diameter and MAP was observed (R^2^ = 0.8928; *p* = 0.05514) (Figure 2C). 

### 3.4. Centhaquine Increases LVOT-VTI (Blood Flow toward Aortic Annulus/Valve)

**Figure 3:**
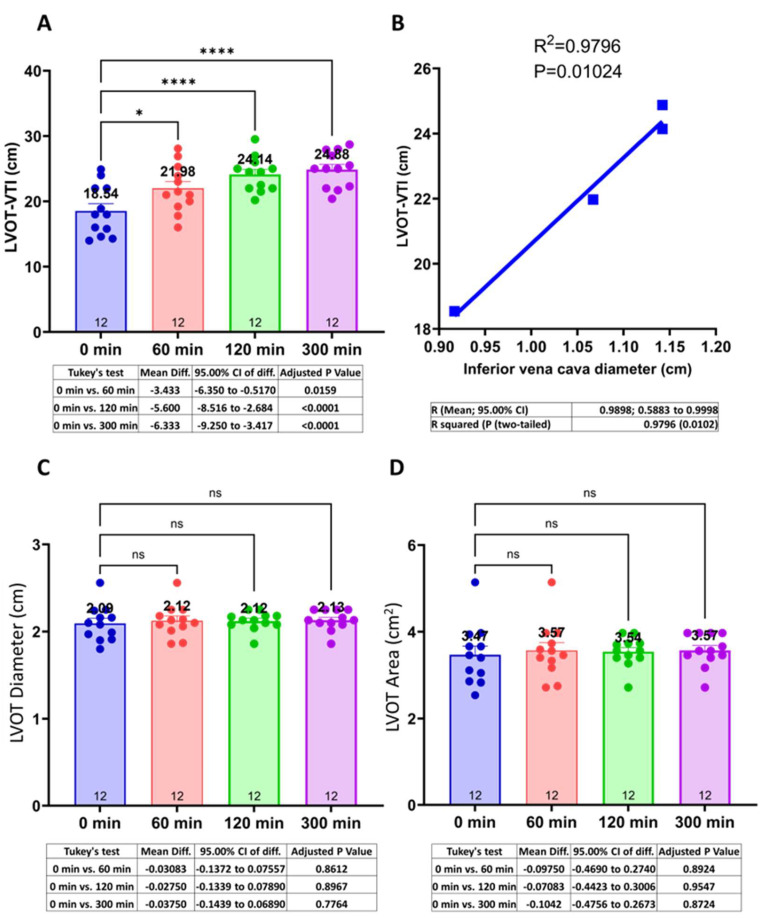
Effect of centhaquine on blood flow (LVOT-VTI) in LVOT to aorta (**A**), its correlation with IVC diameter (**B**), on LVOT diameter (**C**) and LVOT area (**D**). * *p* < 0.05, and **** *p* < 0.0001, ns not significant, compared to 0 min. *n* = 12.

LVOT-VTI (cm) indicates the blood flow from the left ventricle of the heart toward the aorta. LVOT-VTI was 18.54 ± 1.11 at baseline, while it was 21.97 ± 1.05 (Δ18.52%, *p* = 0.0159), 24.14 ± 0.76 (Δ30.2%, *p* < 0.0001), and 24.9 ± 0.8 (Δ34.15%, *p* < 0.0001) at 60 min, 120 min, and 300 min, respectively (Figure 3A). A direct correlation (R^2^ = 0.9796; *p* = 0.01024) between LVOT-VTI and IVC diameter was observed (Figure 3B). The LVOT diameter and area remain unchanged (Figure 3C,D) at these time points. 

## 4. Discussion

Centhaquine increased SV and decreased HR (Figure 1A,C), with a ~40% increase in SV, which is much higher compared to other studies relying on fluid infusion alone (~10–25%) [16,17]. Kumar et al. demonstrated a 15–25% increase in SV in healthy humans after the infusion of 3 L of normal saline at a rate of 1 L per hour and observed cardiac inotropic effects of fluids, with a ~14% increase in LVEF. In the phase II (NCT04056065) clinical trial, resuscitation with centhaquine significantly (*p* = 0.0004) increased MAP compared to patients resuscitated with the control after 24 h (Table 2). Besides SOC, patients were infused with a comparable volume of fluids in both groups (Table 2). These data indicate the resuscitative effects of centhaquine over fluids only (control) in 24 h. 

In our current pilot study, we assessed the fluid volume infused after 5 h of resuscitation and observed that only 746.12 ± 87.42 mL of fluid was required after 5 h (~150 mL per hour) of resuscitation with centhaquine (Table 3) in hypovolemic shock patients, and no change in LVEF and LVFS (Supplementary Figure S1A,B) was seen, indicating no effect of centhaquine on cardiac inotropy. Thus, centhaquine increases SV, reduces the required fluid volume, specifically during the early stages of resuscitation, and does not affect cardiac inotropy. Hence, risks of fluid extravasation and cardiac arrhythmia are mitigated [18], which are common issues associated with a higher volume of fluids and the use of vasopressors to treat shock [19,20]. 

CO and MAP were increased (Figure 1B,D, and Supplementary Table S1) after centhaquine treatment despite reduced HRs and unchanged total peripheral resistance (Supplementary Figure S2), indicating an impact of increased SV on arterial circulation and tissue perfusion. IVC diameter was also significantly increased (Figure 2A), reflecting increased venous return and cardiac preload [21,22,23] after centhaquine treatment. A direct correlation between IVC diameter and SV (R^2^ = 0.9556) (Figure 2B) and between IVC diameter and MAP (R^2^ = 0.8928) (Figure 2C) was observed, which indicated an effect of increased venous return on SV, CO, and MAP. 

Furthermore, LVOT-VTI was significantly upregulated after centhaquine treatment, which indicated increased blood flow in the left ventricular outflow tract during systole, leading to enhanced SV [24,25]. The increased blood flow toward LVOT could be attributed to increased chronotropy, cardiac inotropy, or venous return. After centhaquine treatment, however, patients were observed to have decreased cardiac chronotropy and no change in inotropy (Supplementary Figure S1A,B); hence, increased venous return would be the main reason for the increased LVOT-VTI. The observed direct correlation between IVC diameter and LVOT-VTI (R^2^ = 0.9796; *p* = 0.01024) (Figure 3B) further supports the role of centhaquine-mediated enhanced venous return in improving blood flow toward LVOT and enhancing SV. 

Increased venous return and flow in LVOT would increase the blood volume in the arterial system, leading to increased MAP. Besides blood volume, vascular resistance is also vital for regulating blood pressure. Nonetheless, centhaquine treatment demonstrated no significant change in total peripheral resistance (Supplementary Figure S2), further supporting the role of centhaquine-mediated increased venous return on arterial blood volume, causing an increase in MAP in patients with hypovolemic shock. 

All patients treated with centhaquine in the present study showed improved HR, respiratory rate, and body temperature. A reduction in serum lactate and base deficit and an increase in PO_2_/FiO_2_ were observed in hypovolemic shock patients treated with centhaquine (Table 3). Improved patient outcomes were observed with decreased MODS (0.17 ± 0.11 at the time of discharge vs. 2.5 ± 0.38 at baseline) and stabilized hematological, biochemical, and serum electrolyte levels (Supplementary Table S1). All 12 centhaquine-treated patients recovered and were discharged at 3.1 ± 0.074 days. Thus, the efficacy and safety of centhaquine in hypovolemic shock patients are promising. 

These findings align with studies in animal models of hypovolemic shock [14,26], leading to increased venous return, CO, MAP, and tissue perfusion, and underscore the significance of venous return in managing shock [27], with centhaquine targeting this through α2B-AR agonism. Thus, the current study and other studies [22,23,24] highlight the importance of venous return for treating shock. Venous return is modulated through the venous tone, which is primarily regulated by the sympatho-adrenergic system, and thus, adrenergic signaling appears to be an important target in treating various types of shock [28,29,30,31]. Interestingly, ARs are distributed distinctly in the arterial and venous systems and play a key role in coordinating the arterial and venous circulation. Most arteries and large veins (e.g., vena cava) are mainly regulated by α1/α2-ARs, while peripheral veins are regulated by α2B-ARs [26,32,33,34]. The high abundance of α2B-ARs in the peripheral veins highlights their involvement in the constriction of these peripheral veins. The peripheral veins have relatively higher capacitance than central veins (58.95% vs. 11.05%) and hence are vital players for regulating venous return and cardiac preload, which proportionately affects SV. Therefore, the findings of this study demonstrating increased SV with increased IVC diameter indicate that centhaquine would be acting on the α2B-ARs present in the peripheral veins and inducing venoconstriction, which would mobilize the unstressed blood present in these veins toward the vena cava and lead to increased blood volume in the IVC, causing an increase in its diameter (Figure 2A). However, the current pilot study had several shortcomings, including that this study was non-randomized, it was lacking control patients (SOC + normal saline), and the sample size of the study was small (*n* = 12). 

Moreover, further studies are required to elucidate which specific venous systems are affected after centhaquine treatment. Studies have shown that cutaneous and splanchnic veins, which together constitute the major blood reservoir in the body, respond to various factors, e.g., temperature, stress, arterial blood parameters, and blood pressure [28], and elucidating the effect of centhaquine on the individual venous system would help explore its potential further for the treatment of different types of shock involving circulatory failure in different regions. Moreover, further randomized controlled trials with larger cohorts are necessary to fully understand centhaquine’s effects on venous systems and its potential in treating different types of shock associated with circulatory failure.

## 5. Conclusions

The increased venous return induced by centhaquine plays a pivotal role in elevating SV, CO, and MAP mediated through increased LVOT-VTI and IVC diameter in patients experiencing hypovolemic shock. An increase in SV, CO, and MAP occurs concurrently with a decrease in heart rate, without influencing the inotropic function of the heart. This unique combination of outcomes suggests that centhaquine has remarkable potential to mitigate circulatory failure associated with hypovolemic shock, thereby promoting blood flow and tissue perfusion and improving overall patient outcomes. Understanding centhaquine’s distinctive mechanism of action raises the possibility of its development as a novel resuscitative agent not only for hypovolemic shock but also for other shock types involving circulatory failure and hypotension (e.g., septic shock and vasodilatory shock). 

## Figures and Tables

**Table 1:** **Baseline characteristics of patients**.

Age (years)	37.42 ± 3.68
Body weight (kg)	58.83 ± 1.57
Height (cm)	157.67 ± 1.2
Body mass index (kg/m^2^)	23.64 ± 0.22
**Sex**	
Men	9 (75%)
Women	3 (25%)
**Medical history**	
Hypertension	0
Diabetes	0
Renal disorders	0
Respiratory disease	1 (8.33%)
Ischemic heart disease	0
Liver fibrosis	0
Hepatitis (altered SGPT)	6 (50%)
Pre-eclampsia	0
**Reason for hypovolemic shock**	
Gastroenteritis (vomiting, abdominal pain, and/or diarrhea)	8 (66.67%)
Enteric fever	4 (33.33%)
Dengue fever	2 (16.67%)
Falciparum malaria	1 (8.33%)
Acute appendicitis	1 (8.33%)
**Clinical factors**	
Systolic blood pressure (mmHg)	73.87 ± 2.9
Diastolic blood pressure (mmHg)	45.33 ± 1.64
Heart rate (beats/min)	108 ± 4.4
ECG	Normal
Random blood glucose (mg/dL)	104.24 ± 4.75
Shock index	1.51 ± 0.12
MODS	2.5 ± 0.38
ARDS	0.08 ± 0.083
Respiratory rate (breaths/min)	23.5 ± 0.23
Body temperature (°F)	102.3 ± 0.36
Blood lactate (mmol/L)	2.5 ± 0.06
Base deficit (mmol/L)	−1.49 ± 0.06
Hemoglobin (g/dL)	12.16 ± 0.45
Hematocrit (%)	37.24 ± 1.26
Creatinine (mg/dL)	1.13 ± 0.15
Glomerular filtration rate (mL/min/1.73 m^2^)	83.43 ± 7.84
pH	7.21 ± 0.01
*p*CO_2_ (mmHg)	33.17 ± 0.90
PO_2_/FiO_2_	376.75 ± 6.32

The data are presented as the mean ± SEM.

**Table 2:** Change in MAP and volume of fluid infused in 24 h in hypovolemic shock patients in phase II (NCT04056065) clinical trial.

	Control (*n* = 19)	Centhaquine (*n* = 22)	*p* Value
Change in MAP (mmHg) from 0 (baseline) to 24 h	11.35 ± 2.64	25.09 ± 2.74	0.0004
Volume (Liters) of fluid administered 0 (baseline) to 24 h	2.67 ± 2.64	2.54 ± 0.18	0.3324

The data are presented as the mean ± SEM.

**Table 3:** Volume of fluids administered to patients during first 5 h of resuscitation.

Normal Saline (mL)	Normal Saline with Dextrose (mL)	Total Volume (mL)
345.7 ± 67.2	400.42 ± 32.42	746.12 ± 87.42

The data are presented as the mean ± SEM.

## Data Availability

The anonymized patient data sets generated and/or analyzed during this study are available from the corresponding author on a reasonable request from a bona fide researcher/research group.

## Supplementary Materials

The following supporting information can be downloaded at: https://www.mdpi.com/article/10.3390/jcm13133765/s1, Figure S1: Effects of centhaquine on LVEF and LVFS; Figure S2: Effects of centhaquine on vascular resistance (total vascular resistance); Table S1: Hematological, biochemical, and serum electrolyte levels; Table S2: Study site details.

## Abbreviations

AR	Adrenergic receptor
SV	Stroke volume
CO	Cardiac output
LVEF	Left ventricular ejection fraction
FS	Fractional shortening (left ventricular)
IVC	Inferior vena cava
LVOT	Left ventricular outflow tract
VTI	Velocity time integral
LVDD	Left ventricle diameter at diastole
LVDS	Left ventricle diameter at systole
DBP	Diastolic blood pressure
HR	Heart rate
MAP	Mean arterial pressure
SVR	Systemic vascular resistance
SBP	Systolic blood pressure
SOC	Standard of care
MICE	Multivariate Imputation by Chained Equations
MODS	Multiple Organ Dysfunction Syndrome
ARDS	Acute Respiratory Distress Syndrome
