# Supplementary material for: Centhaquine Increases Stroke Volume and Cardiac Output in Patients with Hypovolemic Shock
Source: J Clin Med. 2024 Jun 27;13(13):3765. doi: 10.3390/jcm13133765 (PMC11242165; doi:10.3390/jcm13133765)
Supplement: Supplementary file 1 [file jcm-13-03765-s001.zip › jcm-3026044-supplementary.pdf]

Supplementary Material

Centhaquine Increases Stroke Volume and Cardiac Output in Patients with Hypovolemic Shock

Aman Khanna[1], Krish Vaidya[2], Dharmesh Shah[3], Amaresh K. Ranjan[4*] and Anil Gulati[4,5,6*]

[1] Aman Hospital and Research Centre Organization, Vadodara, Gujarat, INDIA

[2] I cure Heart care, Vadodara, Gujarat, INDIA

[3] Pharmazz India Pvt. Ltd., Greater Noida, UP, INDIA.

[4]Pharmazz Inc. Research and Development, Willowbrook, IL, USA.

[5]Department of Bioengineering, The University of Illinois at Chicago, Chicago, IL, USA.

[6]Midwestern University, Downers Grove, IL, USA.

***Correspondence:**
anil.gulati@pharmazz.com & amaresh.ranjan@pharmazz.com

Supplementary Figure S1. Effects of centhaquine on LVEF and LVFS. P > 0.05 = ns (not significant) compared to 0 min. n= 12.

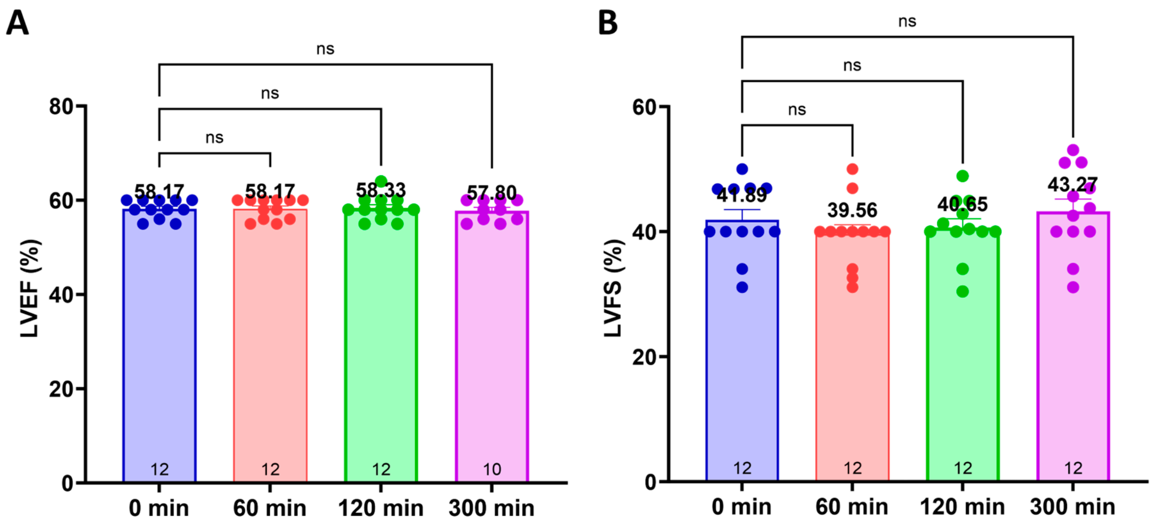

Tukey's test	Mean Diff.	95.00% CI of diff.	Adjusted P Value
0 min vs. 60 min	0.000	-0.9711 to 0.9711	>0.9999
0 min vs. 120 min	-0.1667	-1.138 to 0.8044	0.9660
0 min vs. 300 min	0.1444	-0.8893 to 1.178	0.9811

Tukey's test	Mean Diff.	95.00% CI of diff.	Adjusted P Value
0 min vs. 60 min	2.329	-3.191 to 7.849	0.6672
0 min vs. 120 min	1.242	-4.279 to 6.762	0.9287
0 min vs. 300 min	-1.379	-6.899 to 4.142	0.9056

Supplementary Figure S2. Effects of centhaquine on vascular resistance (total vascular resistance). P > 0.05 = ns (not significant) compared to 0 min. n= 12.

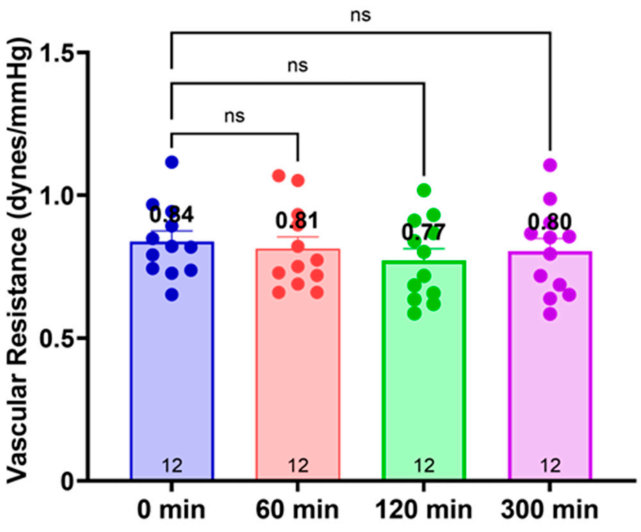

Tukey's test	Mean Diff.	95.00% CI of diff.	Adjusted P Value
0 min vs. 60 min	0.02557	-0.09591 to 0.1470	0.9405
0 min vs. 120 min	0.06575	-0.05573 to 0.1872	0.4700
0 min vs. 300 min	0.03438	-0.08709 to 0.1559	0.8693

Suppl Table S1. Hematological, biochemical, and serum electrolyte levels

	**Day 1 (baseline)**	**Day 3/4**
**Hematology**		
Hemoglobin (g/dL)	12.16 ± 0.45	12.11 ± 0.4
Hematocrit (%)	37.24 ± 1.26	36.7 ± 1.02
Red blood cells ($10^6/mm^3$)	4.60 ± 0.15	4.58 ± 0.13
White blood cells ($/mm^3$)	10692.50 ± 2226.16	9176.01 ± 3097.34
Neutrophils (%)	74.08 ± 3.356	66.50 ± 1.323
Lymphocytes (%)	19.17 ± 2.72	28.00 ± 1.32
Monocytes (%)	5.58 ± 1.11	3.67 ± 0.81
Eosinophils (%)	1.50 ± 0.15	1.83 ± 0.11
Basophils (%)	0.00 ± 0.00	0.00 ± 0.00
Reticulocytes (%)	0.97 ± 0.062	0.90 ± 0.038
Mean corpuscular volume (fL)	81.08 ± 1.687	80.33 ± 1.360
Mean corpuscular hemoglobin (Pg)	26.41 ± 0.512	26.44 ± 0.521
Platelets ($/mm^3$)	163333.33 ± 22663.658	194750.00 ± 16502.123
**Lipid profile**		
Triglyceride (mg/dL)	115.58 ± 6.34	118.58 ± 6.12
Total cholesterol (mg/dL)	166.00 ± 6.314	165.13 ± 5.138
High-density lipoprotein (mg/dL)	46.78 ± 4.335	42.02 ± 0.512
Low-density lipoprotein (mg/dL)	100.28 ± 6.340	99.50 ± 5.176
Very-low-density lipoprotein (mg/dL)	23.12 ± 1.267	23.72 ± 1.218
**Kidney function**		
Serum creatinine (mg/dL)	1.13 ± 0.148	1.01 ± 0.081
Blood urea nitrogen (mg/dL)	16.20 ± 2.115	14.90 ± 1.934
Glomerular filtration rate (ml/min/1.73 m^2)	83.43 ±7.84	95.01 ±7.81
**Liver function**		
Alanine aminotransferase (U/L)	61.74 ± 8.710	47.03 ± 4.163
Aspartate aminotransferase (U/L)	57.43 ± 7.884	43.88 ± 3.989
Serum bilirubin (mg/dL)	0.99 ± 0.064	0.87 ± 0.023
Alkaline phosphatase (IU/L)	119.80 ± 7.691	110.68 ± 5.344
Serum albumin (g/dL)	3.71 ± 0.060	3.65 ± 0.031
Blood glucose (mg/dL)	104.24 ± 4.746	95.32 ± 8.360
**Serum electrolyte**		
Sodium (mmol/L)	138.08 ± 2.113	140.48 ± 1.031
Potassium (mmol/L)	3.96 ± 0.096	4.10 ± 0.054
Calcium (mmol/L)	8.77 ± 0.107	8.82 ± 0.094
**Arterial blood gases**		
pH	7.21 ± 0.01	7.36 ± 0.00
pCO_2 (mmHg)	33.17 ±0.90	37.92 ±1.00

	Day 1 (baseline)	Day 3/4
$pa\mathrm{O_2}$ /$\mathrm{FiO_2}$	376.75 ± 6.321	427.78 ± 2.475

The data are presented as the mean ± SEM. *pH* power of hydrogen, paO_2 partial pressure of oxygen, pCO_2 partial pressure of carbon dioxide, and FiO_2 fraction of inspired oxygen.

Suppl Table S2. Study Site Details

Site No.	Name of Study Site	Name of Ethics Committee	EC Submission Date	EC Approval date
12	Aman Hospital and Research Centre<br>15 Shashwat, Opp, E.S.I Hospital Sarabhai, Gotri Road, Vadodara, Gujarat-390021, India	Institutional Ethics Committee<br>Aman Hospital and Research Centre<br>15 Shashwat, Opp, E.S.I Hospital Sarabhai, Gotri Road, Vadodara, Gujarat-390021, India	07 Nov 2022	30 Nov 2022
